# Supplementary material for: Real-time tracking of the intramolecular vibrational dynamics of liquid water
Source: Commun Chem. 2026 Jan 20;9:57. doi: 10.1038/s42004-025-01863-x (PMC12855949; doi:10.1038/s42004-025-01863-x)
Supplement: Supplementary file 2 — Supplemental material [file 42004_2025_1863_MOESM2_ESM.pdf]

Supplementary Information:

“Real-time tracking of the intramolecular vibrational dynamics of liquid water”

Gaia Giovannetti[1], Sergey Ryabchuk[1,2], Ammar Bin Wahid[1], Hui-Yuan Chen[3], Giovanni Batignani[4],
Erik P. Månsson[1], Oliviero Cannelli[1], Emanuele Mai[4], Andrea Trabattoni[1,5], Ofer Neufeld[6], Angel Rubio[1,7],
Vincent Wanie[1], Hugo Marroux[8,*], Tullio Scopigno[4,*], Majed Chergui[3,9,*], and Francesca Calegari[1,2,10,*]

[1]Center for Free-Electron Laser Science, Deutsches Elektronen-Synchrotron DESY, Notkestr. 85, 22607 Hamburg, Germany

[2]The Hamburg Centre for Ultrafast Imaging, Universität Hamburg, Luruper Chaussee 149, 22761 Hamburg, Germany

[3]Lausanne Centre for Ultrafast Science (LACUS), Ecole Polytechnique Fédérale de Lausanne, ISIC, FSB, Station 6, CH 1015 Lausanne, Switzerland

[4]Dipartimento di Fisica, Università di Roma” La Sapienza”, Roma, I-00185, Italy

[5]Institute of Quantum Optics, Leibniz Universität Hannover, 30167 Hannover, Germany

[6]Schulich Faculty of Chemistry, Technion – Israel Institute of Technology, 32000, Haifa, Israel

[7]Max Planck Institute for the Structure and Dynamics of Matter, Luruper Chaussee 149, 22761 Hamburg, Germany

[8]Laboratoire Interactions, Dynamiques et Lasers, CEA-Saclay, 91191 Gif-sur-Yvette, France

[9]Elettra - Sincrotrone Trieste S.C.p.A., S.S. 14 Km 163, 5 in Area Science Park, I 34149 Trieste, Italy

[10]Physics Department, Universität Hamburg, Luruper Chaussee 149, 22761, Hamburg, Germany

*E-mail: tullio.scopigno@uniroma1.it, hugo.marroux@cea.fr, majed.chergui@elettra.eu, francesca.calegari@desy.de

Supplementary Note 1: Fitting of the pump-probe signal and extraction of the OH stretch mode decay time

The decay time of the OH stretch mode is extracted by fitting the transient signal, which is obtained by integrating the blue side of the differential absorption spectrum between 223 and 253 nm. To consider both the exponential decay and the oscillatory dynamics, the fitting equation is given by:

$$S(t) = a\,(\cos(\omega t + \phi))e^{-bt} + c \qquad (1)$$

where the fitting parameters are:

Parameter	Value	Standard Deviation	Units
a	-0.09	±0.02	mOD
b	0.040	±0.007	fs^{-1}
c	0.00000	$\pm 4.9 \times 10^{-4}$	mOD
ω	0.61	±0.01	rad fs^{-1}
ϕ	1.20	±0.25	rad

Table 1: Fitting parameter values with standard deviations and units

From the parameter b reported in Table 1, we can extract an exponential decay time of 25.0±4.4 fs.

Supplementary Note 2: Laser pulses properties

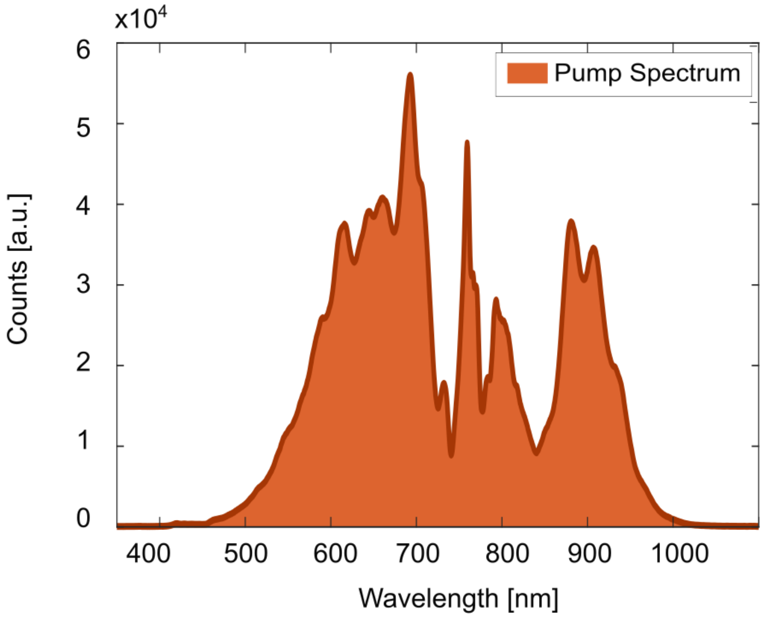

Supplementary Figure 1: Spectrum of the 4.5 fs visible-NIR pump.

Supplementary Figure 1 shows the spectrum of the few-cycle near-infrared (NIR) pump, common to all measurements reported in this work, which spans from 500 nm to 975 nm. The pulse duration is 4.5 fs and it has been measured using the frequency-resolved optical gating (FROG) approach. The spectral and temporal characteristics of the UV probe vary across the different scans. For the scans reported in Fig. 2a, 2b, and 2c, the spectrum of the UV pulses is shown in Supplementary Figure 2 and can support a transform-limited pulse duration of 2.7 fs. For the measurements reported in Figure 4 and Supplementary Figure 7, the chirp of the UV probe is set to C1, C2, and C3 by using a pair of fused silica wedges inserted in the NIR driving laser pulse. The associated spectra are shown in Supplementary Figure 3.

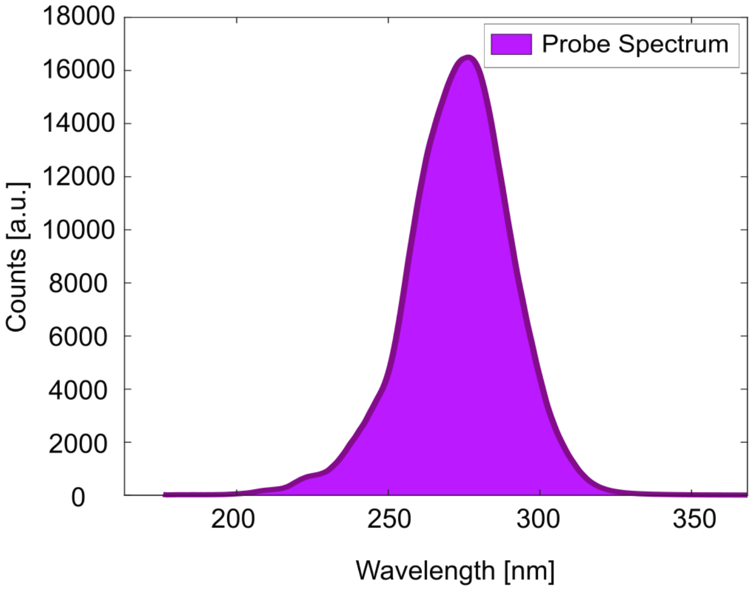

Supplementary Figure 2: Typical UV spectrum used in the experiments. The transform-limited pulse duration is 2.7 fs.

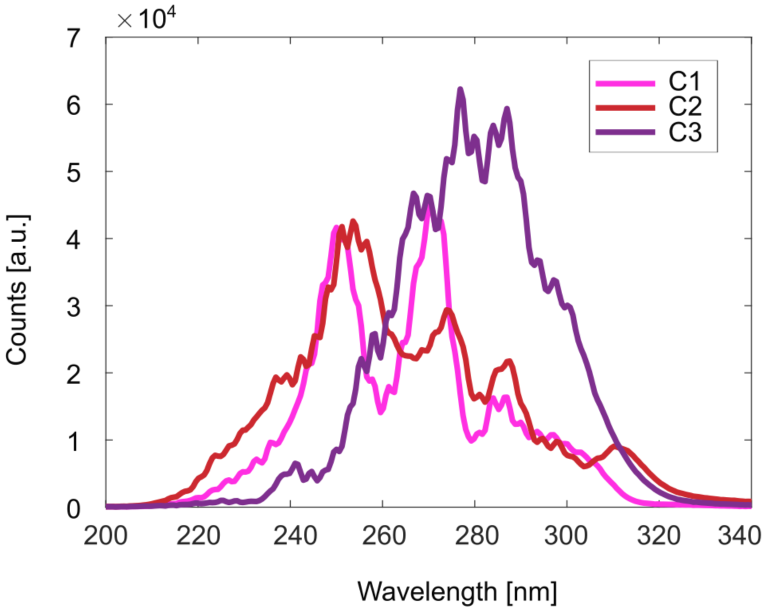

Supplementary Figure 3: UV probe spectra associated with the three chirp values C1, C2 and C3 support transform-limited pulse durations down to 2 fs (as detailed in Supplementary Note 3), but the actual pulse duration is longer due to second order dispersion. The amount of glass inserted in the NIR driver laser used for the UV generation allows for tuning the central wavelength and especially the bandwidth of the UV probe. C2 corresponds to a UV spectrum optimized in terms of bandwidth and spectral phase, while C1 and C3 are obtained by removing and adding glass relative to the optimal wedge position, respectively.

Supplementary Note 3: Retrieval of the probe pulse duration

The time duration and chirp values of the UV pulses associated to the three different spectra in Supplementary Figure 3 have been extracted from the experiment. The upper panels of Supplementary Figures 4, 5, and 6 corresponds to UV static spectra for the three different chirp values of the UV probe: C1, C2, and C3, respectively. These spectra have been recorded after the liquid jet and in absence of the pump pulse.

Single shot UV spectra have been measured for all chirp values and some outlier spectra have been excluded to limit the effects of spectral fluctuations. The remaining shots have then been averaged. The chirp analysis was

conducted fitting the ISRS maps using Eq. 1 reported in the main manuscript (see also Supplementary Note 5 for details) and treating the quadratic phase of the probe pulses as free parameter. The analysis was performed on a restricted region around the central wavelength of the UV spectra, excluding the edges of the spectrum from the fitting process. The overall duration of the UV pulses has been calculated by first determining the transform-limited pulse duration of each averaged spectrum and subsequently adding the quadratic temporal phase extracted from the chirp analysis.

The bottom panels of Supplementary Figures 4, 5, and 6 show the retrieved UV pulse duration for the measurements C1, C2, and C3, respectively, assuming only a second order dispersion. It is worth noting that these results include the dispersive contribution of the water sample, which however does not significantly alter the duration of the transmitted pulses due to the relatively small thickness of the sample (GVD_{water} = 222 fs^2 mm^{-1} at 260 nm, and maximum jet thickness 5 μm).

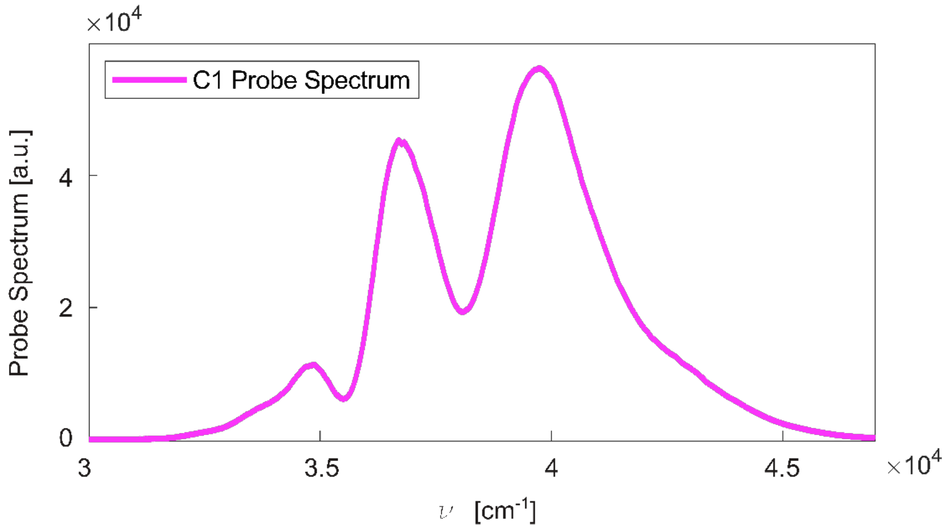

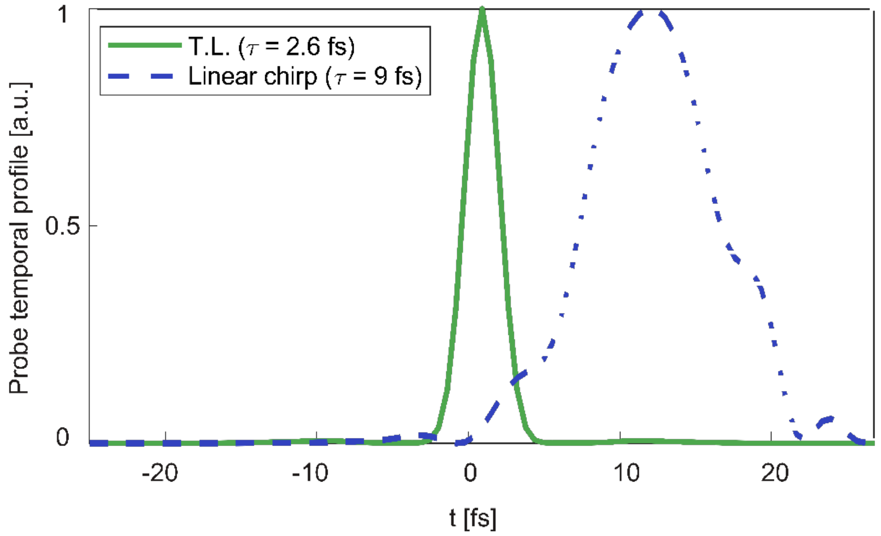

Supplementary Figure 4: Top: Static UV spectrum associated with C1. Bottom: Retrieval of the UV pulse transform-limited duration (green) and of the duration in the presence of linear chirp (blue).

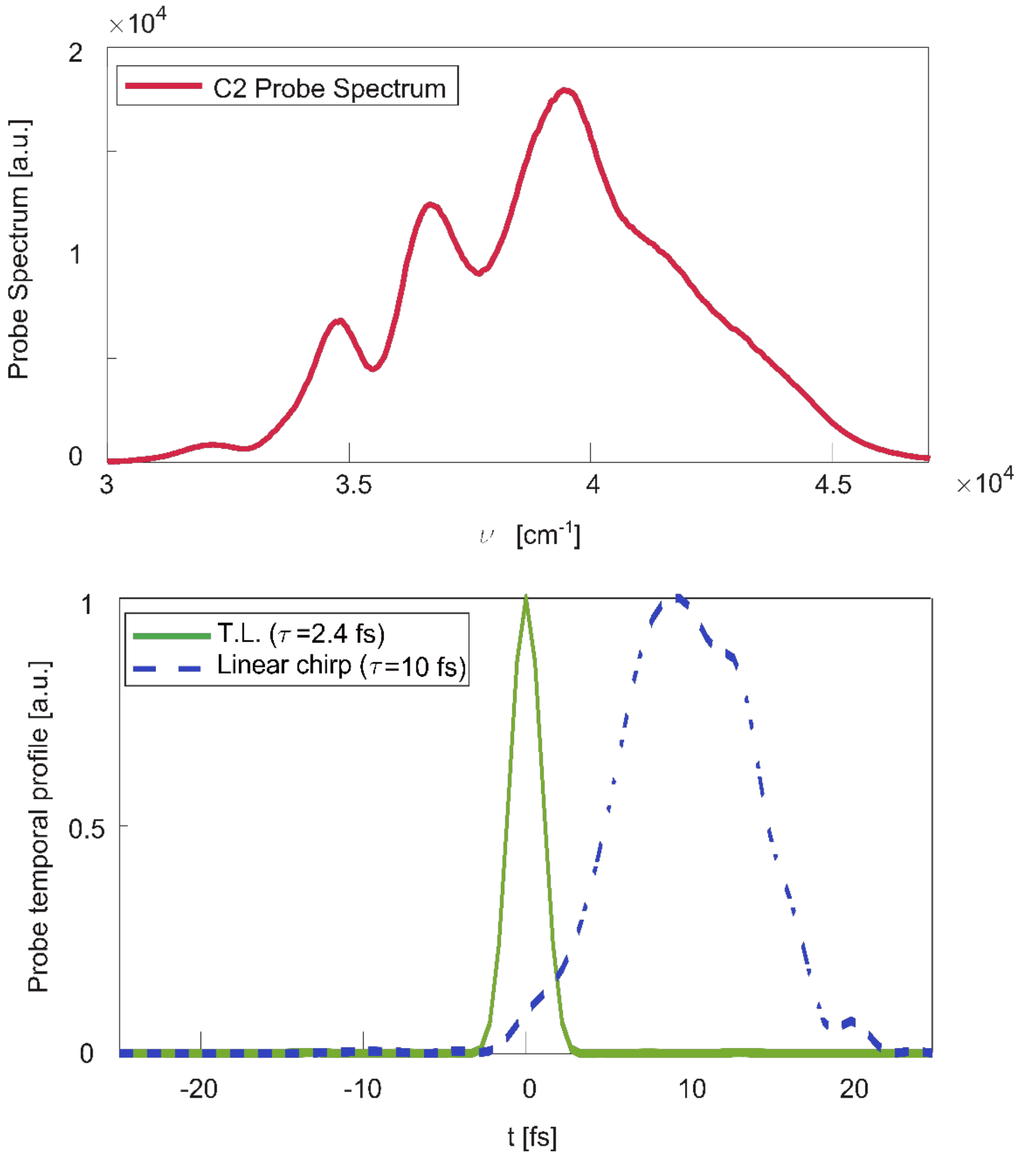

Supplementary Figure 5: Top: Static UV spectrum associated with C2. Bottom: Retrieval of the UV pulse transform-limited duration (green) and of the duration in the presence of linear chirp (blue).

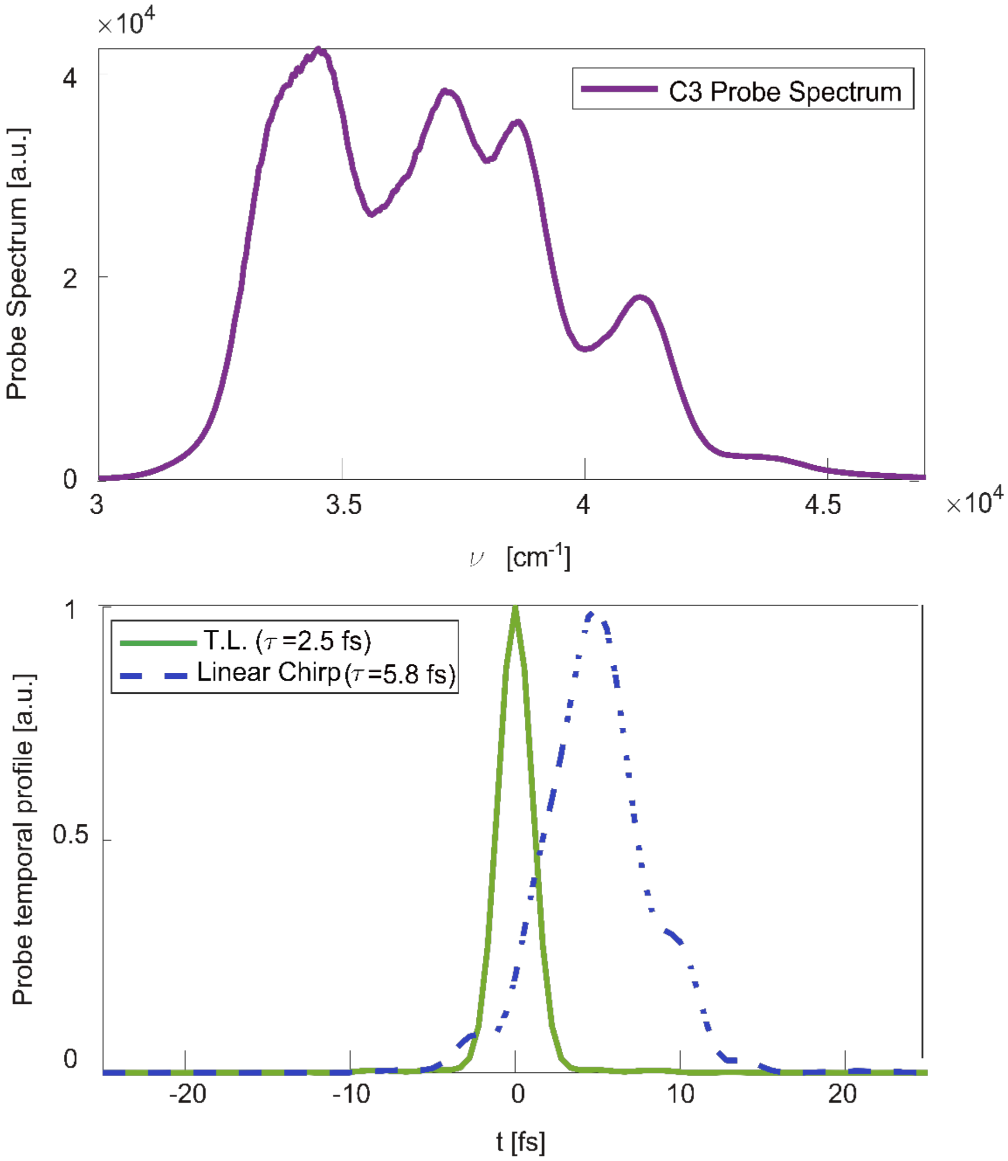

Supplementary Figure 6: Top: Static UV spectrum associated with C3. Bottom: Retrieval of the UV pulse transform-limited duration (green) and of the duration in the presence of linear chirp (blue).

Supplementary Note 4: Fourier Analysis

The Fourier analysis of the scans performed over a time window of 50 fs (Fig. 2c, 4, and Supplementary Figure 7) shows a single dominant feature extending between 3000 and 4000 cm^{-1}. In this case the lower resolution compared to that shown in Fig. 3b and 3c, which were performed over 150 fs, derives from the shorter temporal acquisition window.

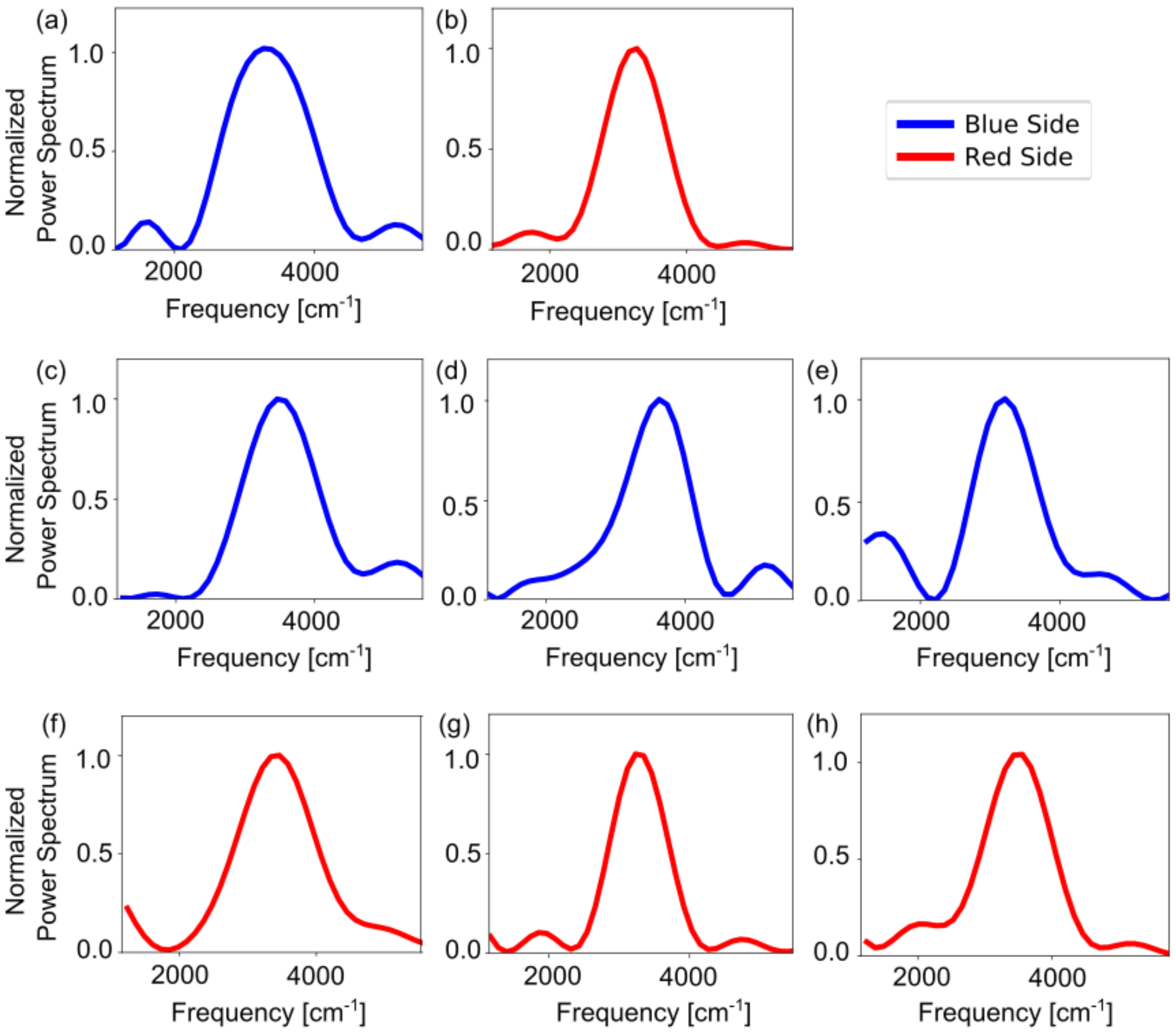

Supplementary Figure 7: Fourier spectra of the blue and red side traces showed in Fig. 2c (a, b) and for the C1 (c, f), C2 (d, g) and C3 (e, h) signals, extracted from the Impulsive Stimulated Raman Scattering (ISRS) data acquired over the short time window of 50 fs and with the same UV probe chirp of the longer scan reported in Fig. 2a and 2b. The transient signals have been integrated on a 30 nm bandwidth (223-253 and 275-305 nm, respectively). The main feature consists in a large band extending between 3000 and 4000 cm^{-1}, corresponding to the OH stretch mode of liquid water.

Supplementary Note 5: Theoretical model

The various pathways contributing to the total spectroscopic response can be depicted using Feynman diagrams[1]. According to the diagrammatic theory[2], the system is represented by the two sides (ket and bra) of the density matrix, while the perturbing electric fields are treated classically[3]. Given the pulse scheme employed in the experiment and the energy levels of liquid water, the two Feynman diagrams illustrating the ISRS response are presented in Figures 2d and 2e. The nonresonant broadband visible-NIR pump, responsible for the first two field interactions at time t_1, initiates a vibrational coherence ($|g'\rangle\langle g|$ or $|g\rangle\langle g'|$) in the ground state of the system via intrapulse Raman excitation. After the perturbation, the system evolves unperturbed until it interacts with the probe at time t_2. As a result, a free induction decay occurs, leaving the system in a population state ($|g'\rangle\langle g'|$ or $|g\rangle\langle g|$), allowing the detection of the vibrational coherence after a variable time delay, T.

The third-order nonlinear polarization responsible for the ISRS process can be evaluated through a perturbative expansion of the molecular density matrix in terms of the electric fields (the Raman pump and the probe). The heterodyne-detected ISRS signal $S(\omega)$ is measured as a function of the probe pulse (P) frequency ω and can be computed from the third-order polarization $P^{(3)}(\omega)$ as:

$$S(\omega) = log\{S'(\omega)\} = log\left\{1 - \Im\left[\frac{P^{(3)}(\omega)E_P^*(\omega)}{I_P(\omega)}\right]\right\} \quad (2)$$

where $E_P(\omega)$ and $I_P(\omega)$ indicate the probe field and intensity, respectively. The symbol * indicates the complex conjugate. To visualize the interference between multiple Raman processes occurring in time-domain vibrational spectroscopy, the total $P^{(3)}(\omega)$ is dissected as the sum of the two A_0 and B_0 diagrams, depending on whether the two interactions with the off-resonant Raman pump involve the ket (generating the $P_A^{(3)}(\omega)$ polarization) or the bra side $P_B^{(3)}(\omega)$) of the density matrix[4]. The total response is obtained as the sum of the A_0 and B_0 contributions, namely $S'(\omega) = S_A(\omega)+S_B(\omega)$. In the case of a fully nonresonant ISRS process, the total response of the system is generated by the interference between the A_0 and B_0 diagrams. This interference occurs between pathways involving interactions with different probe pulse spectral components (red-shifted and blue-shifted relative to the probed wavelength)[4–6] .

In the presence of a chirp in the probe pulse[7], different spectral components reach the sample at varying time delays. This can be modelled by considering a frequency-dependent phase, $\phi(\omega)$, whose derivative at a specific frequency, the group delay $T(\omega) = -\frac{d\phi}{d\omega}$, indicates the arrival time of the corresponding spectral component[8,9]. The probe field $E_P(\omega)$ in the frequency domain can then be written as

$$E_P(\omega) = E_P^0(\omega)e^{i\varphi(\omega)} = E_P^0(\omega)e^{[i\sum_j C_j(\omega-\omega_P)^j]} \quad (3)$$

where $E_P{}^0(\omega)$ is the spectral amplitude and $\phi(\omega)$ has been expanded in powers of the frequency, namely the j-order C_j chirp. The third-order polarization $P_A^{(3)}(\omega)$, which involves the $Q_{g'}$ normal mode, can be calculated from one of the diagrams presented in Figures 2d and 2e as

$$P_A^{(3)}(t) = \left(\frac{i}{\hbar}\right)^2 \frac{\partial\alpha}{\partial Q_{g'}} \mu_{g'e}\mu_{eg} \int_0^\infty d\tau_1 \int_0^\infty d\tau_2 |E_R(t-\tau_1-\tau_2)|^2\, E_P(t-\tau_2) e^{-i\tilde{\omega}_{g'g}\tau_1} e^{-i\tilde{\omega}_{eg}\tau_2} \quad (4)$$

where $\tilde{\omega}_{ij} = \omega_i - \omega_j - i\gamma_{ij}$ and μ_{ij} denotes the dipole transition moment between the i and j states[1,10]. Here, for an electronically off-resonant Raman pulse (R), the radiation-matter interaction Hamiltonian that describes the preparation of the vibrational coherence is given by $H_I^{(R)} = -\alpha \cdot |E_R(t)|^2$, where α indicates the molecular polarizability[11,12]. The interaction Hamiltonian depends on the square modulus of the temporal profile of the pump pulse. The preparation of the vibrational coherence is solely influenced by the intensity profile of the pump and will be represented in the signal as the Fourier transform of this intensity, stressing the importance of the Raman pump pulse duration in ISRS experiments. Short pump pulses are key to impulsively stimulating coherent nuclear wave packets of Raman-active modes. For an ideal Dirac delta temporal profile of the Raman pump, all the nuclear oscillators involved in the Raman-active mode start oscillating in phase, resulting in a strong Raman signal; however, the presence of a temporal chirp introduces a phase difference between the frequency components of the pump participating in the coherence preparation, leading to possible destructive interference. In particular, the longer the Raman pump duration, the greater the phase difference between the nuclear oscillators, reducing the Raman cross section and leading to a vanishing signal. Importantly, if the Raman pump pulse duration is longer than the period T of the Raman-active mode, the vibrational coherence cannot be effectively stimulated[13].

The interaction with the probe pulse leads to a virtual level e, with the interaction Hamiltonian that reads as $H_I^{(P)} = -\mu \cdot E_P$, where μ is the dipole operator. In order to include the inhomogenous broadening, the distribution of vibrational frequencies $G(\omega_v) = G_0(\omega_v - \omega_{g'g})$ can be taken into account as

$$P_A^{(3)}(t) = \left(\frac{i}{\hbar}\right)^2 \frac{\partial\alpha}{\partial Q_{g'}} \mu_{g'e}\mu_{eg} \int_0^\infty d\tau_1 \int_0^\infty d\tau_2 \int_{-\infty}^\infty d\Delta G_0(\Delta)\, |E_R(t-\tau_1-\tau_2)|^2$$
$$E_P(t-\tau_2) e^{-i(\tilde{\omega}_{g'g}+\Delta)\tau_1} e^{-i\tilde{\omega}_{eg}\tau_2} \quad (5)$$

where $G_0(\Delta) = G(\Delta - \omega_{g'g})$ is the spectral inhomogeneous broadening function centred at $\Delta = 0$; by Fourier transforming over Δ it is possible to express the free propagation over τ_1 as a function $\hat{G}_0(\tau)$, which represents the counterpart of the inhomogeneous broadening in the time domain.

This expression for the third order polarization can be exploited to calculate the $S_A(\omega)$ signal as

$$S_A(\omega) = -\Im\left[\frac{E_P^*(\omega)}{I_P(\omega)}\frac{\partial\alpha}{\partial Q_{g'}}\frac{\mu_{g'e}\mu_{eg}}{\hbar^2}\int_{-\infty}^{\infty}d\Delta\int_{-\infty}^{\infty}\frac{d\omega_1}{2\pi}\frac{G_0(\Delta)\hat{I}_R(\omega_1)E_P(\omega-\omega_1)}{(\tilde{\omega}_{g'g}+\Delta-\omega_1)(\tilde{\omega}_{eg}-\omega)}\right] \quad (6)$$

Similarly, for the B diagram we have

$$P_B^{(3)}(t) = -1\cdot\left(\frac{i}{\hbar}\right)^2\frac{\partial\alpha}{\partial Q_{g'}}\mu_{ge}\mu_{eg'}\int_0^{\infty}d\tau_1\int_0^{\infty}d\tau_2\int_{-\infty}^{\infty}d\Delta G_0(\Delta)$$

$$I_R(t-\tau_1-\tau_2)E_P(t-\tau_2)e^{-i(\tilde{\omega}_{gg'}-\Delta)\tau_1}e^{-i(\tilde{\omega}_{eg'}-\Delta)\tau_2}$$

which can be used to calculate the $S_B(\omega)$ signal as

$$S_B(\omega) = +\Im\left[\frac{E_P^*(\omega)}{I_P(\omega)}\frac{\partial\alpha}{\partial Q_{g'}}\frac{\mu_{ge}\mu_{eg'}}{\hbar^2}\int_{-\infty}^{\infty}\frac{d\omega_1}{2\pi}\int_{-\infty}^{\infty}d\Delta\frac{\hat{I}_R^*(\omega_1)E_P(\omega+\omega_1)G_0(\Delta)}{(\tilde{\omega}_{gg'}-\Delta+\omega_1)(\tilde{\omega}_{eg'}-\Delta-\omega)}\right]$$

Expressing the final signal as a function of the pulse delay t_0 yields

$$S_A(\omega,t_0) = -\Im\left[\frac{E_P^*(\omega)}{I_P(\omega)}K\int_{-\infty}^{\infty}\frac{d\omega_1}{2\pi}\int_{-\infty}^{\infty}d\Delta\frac{G_0(\Delta)\hat{I}_R^{(0)}(\omega_1)E_P(\omega-\omega_1)e^{-i\omega_1 t_0}}{(\tilde{\omega}_{g'g}+\Delta-\omega_1)(\tilde{\omega}_{eg}-\omega)}\right] \quad (7)$$

and

$$S_B(\omega,t_0) = +\Im\left[\frac{E_P^*(\omega)}{I_P(\omega)}K\int_{-\infty}^{\infty}\frac{d\omega_1}{2\pi}\int_{-\infty}^{\infty}d\Delta\frac{G_0(\Delta)\hat{I}_R^{(0)*}(\omega_1)E_P(\omega+\omega_1)e^{+i\omega_1 t_0}}{(\tilde{\omega}_{gg'}-\Delta+\omega_1)(\tilde{\omega}_{eg'}-\Delta-\omega)}\right] \quad (8)$$

where

$$K = \frac{\partial\alpha}{\partial Q_{g'}}\frac{\mu_{ge}\mu_{eg'}}{\hbar^2}.$$

These equations can be exploited to directly calculate the ISRS response as a function of both the molecular and experimental parameters. It is worth to stress that for an electronically off-resonant probe with transform-limited pulses (C_j=0) and a flat probe spectral profile, these two signals generate time oscillations with the same amplitude but opposite phase; under these conditions, S_A and S_B interfere destructively, suppressing the ISRS signal[5,14]. The π phase difference between the two signals can be interpreted as a result of the fact that the preparation of the vibrational coherence occurs on the ket side of the density matrix for the A_0 diagram, and on the bra side for the B_0 diagram. As a result, the vibrational coherence described in the two diagrams form a complex conjugate pair ($|g\rangle\langle g'| = |g'\rangle\langle g|^*$). Interestingly, the signal measured at a given probe frequency ω is generated by two pathways which involve spectral components of the probe that are red- (S_A) and blue-shifted (S_B) by approximately one vibrational quantum. For this reason, the amplitudes of the S_A and S_B terms are generally different for a non-flat probe spectral profile. Most importantly, introducing a chirp in the probe pulses allows tuning of the relative phase between the S_A and S_B processes, thereby ensuring an amplification of the measured signal.

Supplementary Note 6: Spontaneous Raman spectral components

Table 2 reports the values of the Raman shift, FWHM and amplitude of the three Gaussian components used to fit the OH stretch band of the spontaneous Raman spectrum of liquid water[15]. Although there is no general

consensus about the specific number of gaussian components to be used to fit the OH stretch Raman band, a minimal model based on three components can be used to properly describe both spontaneous Raman and time-domain ISRS spectra. These values are used as constrained parameters to fit the ISRS maps.

**Raman shift (cm^{-1})**	**FWHM (cm^{-1})**	**Amplitude (AU)**
3259	275	0.83
3447	179	0.71
3593	159	0.31

Table 2: Input parameters of the fit of the ISRS maps, extracted from the fit of the OH stretch band in Spontaneous Raman measurements.

The chirp parameters obtained from the fit of the ISRS maps C1, C2 and C3 are reported in Table 3 and 4 for linear or higher order chirp, respectively:

	**First order [fs^2]**	**Second order [fs^3]**	**Third order [fs^4]**	**Fourth order [fs^5]**	**t_0 [fs]**
**C1**	-3.8	0	0	0	-3.8
**C2**	4.2	0	0	0	7.5
**C3**	2.8	0	0	0	3.3

Table 3: Parameter extracted by the fit of the ISRS maps for the C1, C2 and C3 conditions with linear chirp, while t_0 is a time offset.

	**First order [fs^2]**	**Second order [fs^3]**	**Third order [fs^4]**	**Fourth order [fs^5]**	**t_0 [fs]**
**C1**	-2.9	-3.0	-2.8	-0.2	-2.5
**C2**	5.5	-1.7	-2.5	-0.1	8.3
**C3**	1.1	-1.1	2.8	2.3	3.3

Table 4: Parameter extracted by the fit of the ISRS maps for the C1, C2 and C3 conditions with fourth-order chirp, while t_0 is a time offset.

Supplementary Note 7: ISRS pump intensity dependence

Supplementary Figure 8 shows the Fourier transform amplitude of the time-domain pump-probe signal as a function of the pump intensity. The data are in good agreement with the linear fit (dashed line) confirming the ISRS origin of the signal due to ground state vibrations of water molecules, and excluding multiphoton absorption processes.

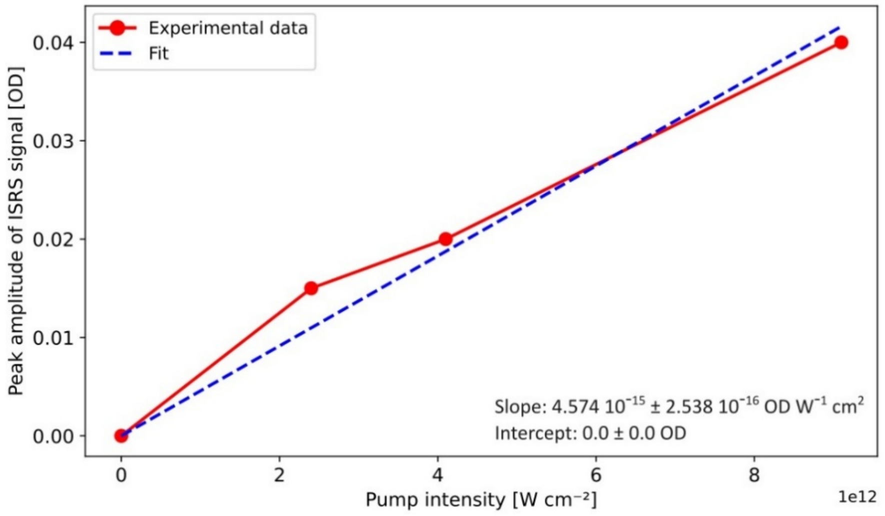

Supplementary Figure 8: Dependence of the ISRS signal to the pump intensity. The dashed line corresponds to the linear fit of the experimental data.

Supplementary Note 8: Pump-probe signal at early time delays

Supplementary Figure 9 shows the color maps of the early delay times for the C1, C2 and C3 cases. The coherent artifact is fitted by a fifth-order polynomial (dashed yellow line). The FWHM of the coherent artifact does not give a good estimate of the instrument response function (IRF). In fact, in frequency-resolved measurements the possibility of observing coherent oscillations is limited by the pump pulse duration but, in view of the spectrally dispersed detection, it is not compromised by non-transform-limited probe pulses[16], whereas the FWHM of the coherent artifact reflects the effective pulse duration of the probe, which is broadened by the chirp.

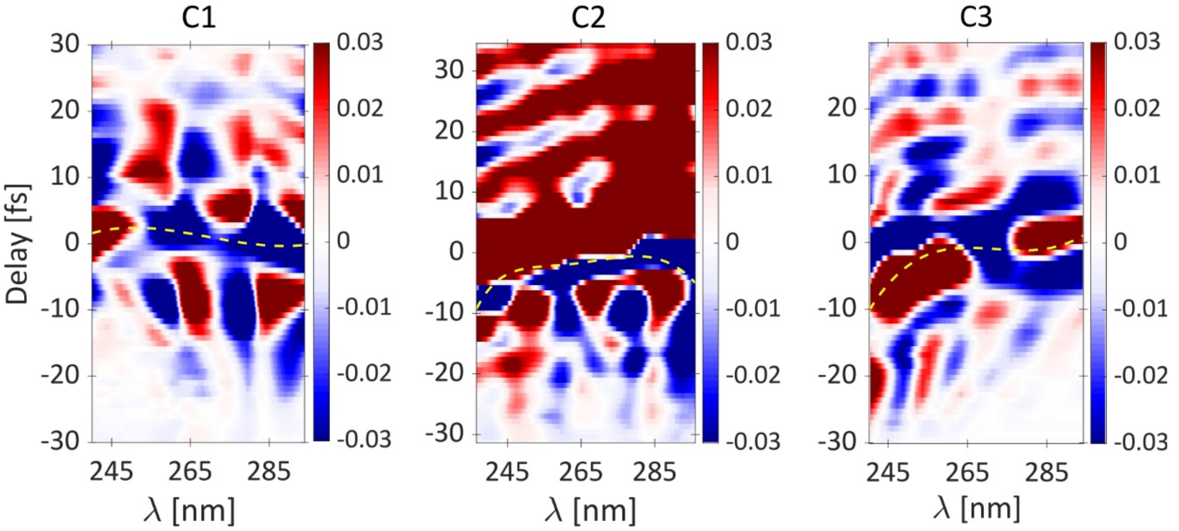

Supplementary Figure 9: Colormaps at early times for the C1, C2 and C3 chirp values. The coherent artifact is fitted by a polynomial curve (dashed yellow line).

Supplementary References

1. Batignani, G., Fumero, G., Mukamel, S. & Scopigno, T. Energy flow between spectral components in 2D broadband stimulated Raman spectroscopy. *Phys. Chem. Chem. Phys.* **17**, 10454–10461 (2015).
2. Mukamel, S. *Principles of Nonlinear Spectroscopy*. (Oxford University Press, New York, 1995).

3. Mukamel, S. & Rahav, S. Ultrafast Nonlinear Optical Signals Viewed from the Molecule's Perspective. in *Advances In Atomic, Molecular, and Optical Physics* vol. 59 223–263 (Elsevier, 2010).
4. Batignani, G., Ferrante, C., Fumero, G. & Scopigno, T. Broadband Impulsive Stimulated Raman Scattering Based on a Chirped Detection. *J. Phys. Chem. Lett.* **10**, 7789–7796 (2019).
5. Gdor, I., Ghosh, T., Lioubashevski, O. & Ruhman, S. Nonresonant Raman Effects on Femtosecond Pump–Probe with Chirped White Light: Challenges and Opportunities. *J. Phys. Chem. Lett.* **8**, 1920–1924 (2017).
6. Zhou, Y., Constantine, S., Harrel, S. & Ziegler, L. D. The probe frequency dependence of nonresonant femtosecond pump–probe nuclear responses: Undercutting vibrational inhomogeneities. *J. Chem. Phys.* **110**, 5893–5905 (1999).
7. Agrawal, G. *Nonlinear Fiber Optics*. (Academic Press, 2013).
8. Walmsley, I. A. & Wong, V. Characterization of the electric field of ultrashort optical pulses. *J. Opt. Soc. Am. B* **13**, 2453 (1996).
9. Smith, B. J., Mahou, P., Cohen, O., Lundeen, J. S. & Walmsley, I. A. Photon pair generation in birefringent optical fibers. *Opt. Express* **17**, 23589 (2009).
10. Dorfman, K. E., Fingerhut, B. P. & Mukamel, S. Time-resolved broadband Raman spectroscopies: A unified six-wave-mixing representation. *J. Chem. Phys.* **139**, 124113 (2013).
11. Tanimura, Y. & Mukamel, S. Two-dimensional femtosecond vibrational spectroscopy of liquids. *J. Chem. Phys.* **99**, 9496–9511 (1993).
12. Batignani, G., Mai, E., Fumero, G., Mukamel, S. & Scopigno, T. Absolute excited state molecular geometries revealed by resonance Raman signals. *Nat. Commun.* **13**, 7770 (2022).
13. Batignani, G. *et al.* Excited-State Energy Surfaces in Molecules Revealed by Impulsive Stimulated Raman Excitation Profiles. *J. Phys. Chem. Lett.* **12**, 9239–9247 (2021).
14. Monacelli, L. *et al.* Manipulating Impulsive Stimulated Raman Spectroscopy with a Chirped Probe Pulse. *J. Phys. Chem. Lett.* **8**, 966–974 (2017).
15. Choe, C., Lademann, J. & Darvin, M. E. Depth profiles of hydrogen bound water molecule types and their relation to lipid and protein interaction in the human stratum corneum in vivo. *The Analyst* **141**, 6329–6337 (2016).
16. Polli, D., Brida, D., Mukamel, S., Lanzani, G. & Cerullo, G. Effective temporal resolution in pump-probe spectroscopy with strongly chirped pulses. *Phys. Rev. A* **82**, 053809 (2010).
